# “Without it, I am not sure I would still be here”: a mixed methods service evaluation for online EMDR trauma therapy in a primary care network in England

**DOI:** 10.3389/fpsyt.2023.1301540

**Published:** 2023-11-28

**Authors:** Safa Kemal Kaptan, Carsten Dernedde, Tilda Dowden, Ayşe Akan

**Affiliations:** ^1^Department of Psychology, Boğaziçi University, Istanbul, Türkiye; ^2^Division of Psychology and Mental Health, Institute of Teaching and Learning, University of Manchester, Manchester, United Kingdom; ^3^Hoveton and Wroxham Medical Centre, Norwich, United Kingdom; ^4^Norwich Medical School, University of East Anglia, Norwich, United Kingdom; ^5^Norfolk and Suffolk NHS Foundation Trust, Norwich, United Kingdom; ^6^North East London NHS Foundation Trust, London, United Kingdom

**Keywords:** EMDR therapy, trauma, primary care, general practice, mental health, service development, online EMDR, service evaluation

## Abstract

**Introduction:**

Psychological services are typically offered via specialized mental health services, which are often overwhelmed with long waitlists. To address this need and provide patients with a service characterized by shorter waiting times and increased accessibility, online Eye Movement Desensitization and Reprocessing (EMDR) was established in the North Norfolk 4 Primary Care Network.

**Methods:**

This article presents this service’s collaborative funding, development and outcomes within local GP surgeries. It constitutes a mixed-method service evaluation encompassing the future of EMDR in primary care services. Additionally, it includes the assessment of anxiety, depression, and PTSD symptoms as well as work and social adjustment in a cohort of 83 patients alongside a Thematic Analysis involving eighteen patients and six GPs.

**Results:**

The evaluation showed high completion and attendance among service users. Quantitative scores combined with qualitative feedback from patients and practitioners highlight the potential impact of EMDR therapy on General Practice and its broader provision of trauma-focused therapies. The most significant improvements were observed in anxiety and depression scores. Thematic Analysis indicated that the patients found the service helpful, labeling it as a “life-saver.” They also discussed why they found the service effective; some also wished the service had been available earlier.

**Discussion:**

Findings underscore the potential of EMDR and online EMDR as an accessible and effective approach within primary care settings. The assessments showed an elevated level of access and attendance among service users. Therefore, it is recommended that timely EMDR services be extended through primary care networks.

## 1 Introduction

The North Norfolk 4 Primary Care Network (NN4 PCN) is a consortium of six GP Practices in the UK: Acle Surgery, Blofield Surgery, Brundall Surgery, Hoveton and Wroxham Medical Centre, Ludham Surgery, and Stalham Staithe Surgery. NN4 PCN operates within a semi-rural setting in Norfolk and caters to an approximate patient population of 50,000 individuals. Within the region, there seems to be recognition and wide acknowledgment of the impact of trauma and trauma-informed care. Consequently, the availability of trauma therapies is increasing.

Norfolk and Suffolk NHS Foundation Trust offers trauma therapy at the primary care level through the Wellbeing Service (previously IAPT) and at the secondary care level, albeit at a limited capacity. Primary care services, facilitated by General Practitioners (GPs) and other healthcare providers, offer accessible, comprehensive, and ongoing care to individuals and communities. On the other hand, secondary care focuses on specialized and in-depth care. Typically, it requires a referral from a primary care provider and may involve hospital-based or community-based services. In practice, however, there appeared to be a service gap for patients for several reasons. These factors encompass, among others, limited funding and a national shortage of trained mental health professionals (1–3) as well as a further surge in demand for mental health services due to COVID-19. In secondary care, for example, referred patients are typically offered an initial assessment within the first 3–6 months after their referral. Following this assessment, if directed to psychology services, they may face an additional 6–12 months’ wait to be seen by a psychological practitioner, posing challenges for timely interventions (4). Consequently, patients endure year-long waits in two-thirds of cases (5) which negatively impacts future attendance rates and making individuals resistant to services (6, 7). While waiting times can be shorter in some services, Norfolk typically also reports waiting times ranging from several weeks to several months. For example, a recent report suggests that more than 4,000 children are on waiting lists for mental health assessments, with over 1,000 waiting for longer than a year (8). Finally, mental health services are predominantly built around cognitive-based therapies and other approaches are offered as an “add-on” only if psychologists trained in that approach happen to be recruited and the Trust can arrange specialized supervision. Due to the limited availability of other forms of therapies, even when the first line of treatment does not benefit a patient in a meaningful way or has limited benefits, the secondary mental health services may discharge the patient back to their GPs. Yet, GPs are unable to decide to add a specific therapy in their primary care and secure funding for it, given the NHS does not employ them; instead, they are independent contractors.

In 2019, NHS England introduced funding to Primary Care Networks (PCNs) through the Additional Roles Reimbursement Scheme (ARRS) to enhance the primary care workforce. The funding allowed GP practices to recruit Mental Health Practitioners to establish further services in their primary care by offering first assessment, brief intervention and/or liaison with mental health services for patients with mental health difficulties.

Taking advantage of the funding, The North Norfolk 4 Primary Care Network (NN4 PCN) proposed to use this funding innovatively by locating highly effective psychotherapy, in the form of Eye Movement Desensitization and Reprocessing (EMDR), directly within GP Practices. The idea of creating such a service directly within GP Practices was very well received by the members of NN4 PCN, who were dissatisfied with the accessibility of trauma-focused therapies. The research team’s prior engagements and discussions with General Practitioners (GPs) revealed that EMDR in GP practices was a promising option. The noteworthy accomplishments of ARC, a counselling charity affiliated with a GP practice in Axminster, were also a significant inspiration. The charity served as a notable model of effective EMDR integration within primary care, having been in operation since 2007 and assisting more than 2,500 patients.

As a result, the Primary Care Network adopted the proposal to establish EMDR service to address the perceived service gap and provide patients with a responsive service characterized by shorter waiting times, offered by their local GP surgery, tailored to their unique needs and most importantly, effective. The local Mental Health Trust, Norfolk and Suffolk Foundation Trust (NSFT), generously decided to support the project by seconding the full-time EMDR Consultant who had expressed an interest in the post.

The present article aims to evaluate the feasibility of this service and its potential in primary care services. In addition, the study aims to determine whether online EMDR therapy effectively alleviates clinical symptoms associated with common mental health conditions.

## 2 Methods

### 2.1 Design

The current article is a service evaluation, employing a mixed-method approach combining quantitative and qualitative methods. Quantitative measures were utilized to evaluate potential changes in outcome scores over time, while a qualitative questionnaire was used to gain insight into patients’ experiences and viewpoints regarding their involvement in the intervention.

### 2.2 Procedure and referrals

The EMDR therapist worked across the six GP practices within NN4 PCN. Serving as an integral member of each practice’s team, the therapist facilitated patient referrals through internal communication channels.

The referral criteria were written to ensure simplicity and efficacy. They were formulated to identify patients with limited prospects of receiving therapy from alternative sources within the NHS or the voluntary sector. Moreover, the criteria aimed to identify patients who were most likely to benefit from EMDR therapy.

All referrals were generated from within the member practices of the PCN. Only patients registered with one of the member practices were referred. Only GPs and other clinicians in the practice team (Advanced Nurse Practitioners, GP trainees, MH practitioners, MH and Wellbeing Coaches) were able to refer. As the EMDR therapist was a practice team member of each practice, no referral letter was needed. The therapist had access to the patient record to see the patient’s symptoms and trauma history. The EMDR therapist could return the patient’s progress to the referrer. Thanks to the single shared patient record, administrative time was minimized and time available for therapy was maximized.

The referral criteria were designed to be easily understandable and not dependent on a formal diagnosis of PTSD. No limitation was placed on the severity of presenting symptoms, but there had to be a realistic prospect that therapy could be completed in 4 to 8 sessions. That meant that patients with high complexity and low resources, particularly in childhood trauma or neglect cases, needed to be referred elsewhere. Other exclusions are patients who can be referred to other services in the statutory or voluntary sector, such as military veterans or survivors of sexual abuse.

### 2.3 Sample

The inclusion criteria for referral to the EMDR therapy service encompassed a range of circumstances related to traumatic events and their consequences. These included presentations characterized by nightmares, flashbacks, hypervigilance, reactive anxiety and depression, bereavement, separation, domestic violence, job loss, COVID-19 related difficulties, traffic accidents, sexual assault in adulthood and cases where there was either an established diagnosis or a potential for the development of post-traumatic stress disorder (PTSD).

On the other hand, the exclusion criteria guide the referral process by identifying cases that may be better suited for alternative services. These include the risk of self-harm or suicide attempts, somatization and dissociation, chronic pain, myalgic encephalomyelitis (ME), or fibromyalgia. Patients currently undergoing legal proceedings or compensation claims, survivors of rape requiring legal support, ongoing trauma, obsessive-compulsive disorder (OCD), patients under 16 years of age, substance use disorders, combat trauma, eating disorders and those currently engaged in therapy elsewhere were excluded and referred to appropriate services based on their specific needs.

### 2.4 The intervention

EMDR therapy is a psychotherapeutic approach based on the Adaptive Information Processing (AIP) model (9, 10). The AIP model states that the human brain naturally assimilates and integrates new experiences, making them functional. However, when individuals experience an elevated level of stress, their processing system gets “stuck” and experiences are stored in a dysfunctional state, leading to distress. The standard EMDR protocol comprises eight phases designed to mitigate the negative impact of unprocessed memories (11). These phases encompass history taking, preparation, assessment, reprocessing and desensitization of distressing memories, installation of positive cognitions, a body scan, closure, and re-evaluation. What sets EMDR apart from other psychological therapies is the reprocessing phase. In EMDR, this phase incorporates a unique set of techniques known as “bilateral stimulation” (BLS), which typically involves guiding clients through sequences of eye movements. At the same time, they focus on a troubling memory and its associated thoughts, emotions and bodily sensations (10).

Numerous studies have indicated that EMDR effectively reduces symptoms of mental health difficulties. These studies encompass both clinical and nonclinical populations, including those dealing with PTSD (12, 13), depression (14), and anxiety (15). As a result of this growing body of evidence of effectiveness, EMDR has gained strong recognition and endorsement as an effective treatment for PTSD from several influential organizations. These include the American Psychiatric Association (16), the World Health Organization (17), the National Institute for Health and Care Excellence (18), and the International Society for Traumatic Stress Studies (19).

In the current project, the standard EMDR protocol was utilized within a relatively brief timeframe (4 to 8 sessions), with sessions exclusively conducted by an EMDR Consultant remotely via Zoom. This has been shown to be as effective as face-to-face treatment (20, 21). It was also practically advantageous because the PCN covers a large and rural geographical area which means that the therapist did not need to travel between six practices every week and could offer the travel time as sessions to other patients and the patients could also benefit from not needing to commute. It also has the added advantage for PCN member practices: the service provision does not impact the shortage of consulting rooms that all practices experience.

### 2.5 Outcome measures

If the Primary Care Service did not offer EMDR, patients would likely be triaged to the Wellbeing Service. For this reason, the same psychometrics the Wellbeing Service use was employed to ensure consistency in outcome measures, namely the Patient Health Questionnaire-9 (PHQ-9), General Anxiety Disorder-7 (GAD-7), and Work And Social Functioning (W&SAS). In addition, due to the trauma focus the PTSD Checklist for DSM-5 (PCL-5) to screen for PTSD symptoms was included.

*The Patient Health Questionnaire-9 (PHQ-9)* (22) is a nine-item scale to measure the severity of depressive symptoms. The total score ranges from 0 to 27. The PHQ-9 consists of five categories, with the following cut-off points indicating different levels of depressive symptoms: 0–4 signifies no depressive symptoms, 5–9 indicates mild depressive symptoms, 10–14 suggests moderate depressive symptoms, 15–19 reflects moderately-severe depressive symptoms, and 20–27 signifies severe depressive symptoms. The PHQ-9 has a high degree of internal consistency (0.88) (23).

*The Generalized Anxiety Disorder 7-item (GAD-7)* (24) has 7 items with cut-offs of 5, 10, 15, indicating mild, moderate, and severe anxiety levels, respectively. GAD-7 has a robust internal consistency of 0.89 for the general population (25).

*The Work and Social Adjustment Scale (W&SAS)* (26) is a 5-item assessment tool to measure functional impairment. It employs an eight-point Likert scale and a value of 20 or higher suggests moderately severe functional impairment while a score ranging from 10 to 20 suggests a relatively milder functional impairment. The scale has an internal scale consistency ranged from 0.70 to 0.94 (26).

*The PTSD Checklist (PCL-5)* (27) following DSM-5 guidelines, is a twenty-item scale to evaluate symptoms of post-traumatic stress disorder (PTSD). Each item measures the intensity of a specific symptom on a five-point Likert scale ranging from 0 to 4 for each symptom. A cutoff score between 31 and 33 is considered a probable PTSD across various samples. PCL-5 scores exhibited strong internal consistency (α = 0.94) (27).

Upon completion of the intervention, patients and their GPs were invited to complete online questionnaires to explore their experiences and perceptions with the intervention. The online questionnaires included questions on the intervention including content, delivery method, usefulness, acceptability and barriers or facilitators to implementation.

### 2.6 Data analysis

As this is a service evaluation, no formal hypothesis testing was undertaken. The study was not powered to assess group differences. The study aims to demonstrate a trend in favor of the intervention and the recruitment and retention of patients into the study. Descriptive statistics along with corresponding measures were reported and quantitative data were analyzed using Reliable Change Index (RCI) and clinically significant change (CSC) (28). The Reliable Change Index (RCI) and Clinically Significant Change (CSC) are used to determine whether an individual’s change is statistically reliable and clinically meaningful (29).

The RCI assesses whether the score change between two-time points for an individual on a measure is larger than measurement error alone. In other words, it helps determine if a score change is statistically significant.

The CSC goes beyond statistical significance to assess whether the score change is meaningful from a clinical perspective. The CSC asks whether the change is enough to make a meaningful difference in the individual’s life. To meet the CSC criterion, an individual’s change should not only be statistically reliable (as indicated by the RCI) but also should result in the individual moving from being classified as “impaired” or “unhealthy” to a range considered “healthy” or “functional,” on the measure.

The qualitative data was analyzed using Thematic Analysis (30, 31). Thematic Analysis begins with researchers familiarizing themselves with the data, identifying meaningful patterns (codes), grouping these codes into potential themes, refining and defining the themes, and interpreting the data for analysis. This method offers a structured approach to understand and interpret insights within qualitative data. Philosophically, the researchers adopted a “critical realist” approach (32, 33). The analysis was conducted inductively, primarily focusing on manifest meaning, while also exploring latent meaning (34).

## 3 Findings

### 3.1 Sample characteristics

Out of the 139 patients who were referred, 83 were provided with EMDR treatment, and 56 of them received consultation. This consultation involved directing patients to other services. However, 19 patients did not actively participate in the process.

The patients had an average age of 44 years, and out of the total, 99 were identified as female. Patients were referred for several reasons, including physical and sexual assault, childhood abuse and neglect, loss and bereavement, medical difficulties, traumatic experiences at work, domestic violence, and social difficulties (Table 1).

**Table 1:** Participants’ demographic characteristics.

		*N* (%)
Gender	Female	99
	Male	40
Age (years)	14–18	8
	19–30	29
	31–40	24
	41–50	30
	51–60	25
	61–70	14
	71+	9
Reason for referral	Physical and sexual assault	30
	Childhood trauma- low complexity / risk	25
	Loss and bereavement	23
	Medical difficulties	21
	Traumatic experiences at work	16
	Domestic violence	12
	Social difficulties	12

There were 139 referrals across all surgeries, consisting of fifty-six consultation-only cases and 83 patients offered EMDR (Table 2).

**Table 2:** Referral numbers based on surgeries.

Surgery	Referrals	Consultation	Offered EMDR
Acle	20	9	11
Blofield	34	14	20
Brundall MP	8	3	5
Hoveton and Wroxham MC	39	14	25
Ludham and Stalham Green	23	10	13
Staithe surgery	15	6	9
Total	139	56	83

### 3.2 Clinical outcomes

Although this study did not aim to measure clinical effectiveness using a randomized controlled trial, the results indicated improvements on all scales. The quantitative data were analyzed using RCI and CSC analyses. To calculate RCI and CSC, the mean scores of each tool at each data collection point and standard deviations were used. The results are outlined below (Table 3).

**Table 3:** Quantitative results.

	GAD-7	PHQ-9	PCL-5	W&SAS
Pre-treatment scores mean (SD)	14.51 (3.74)	16.15 (5.26)	46.05 (12.33)	19.55 (8.24)
Post-treatment scores mean (SD)	4.38 (3.41)	5.53 (4)	11.20 (8.39)	8.15 (6.41)
PRE-post effect size	2.71	2.02	2.83	1.38
Reliable change index value	3.28	5.83	13.67	9.14
Number “no change”	3	10	2	23
Number “deteriorate”	0	0	0	0
Number “improved”	51	44	52	30
Number meeting clinically significant change	50	44	39	30

**Figure 1:**
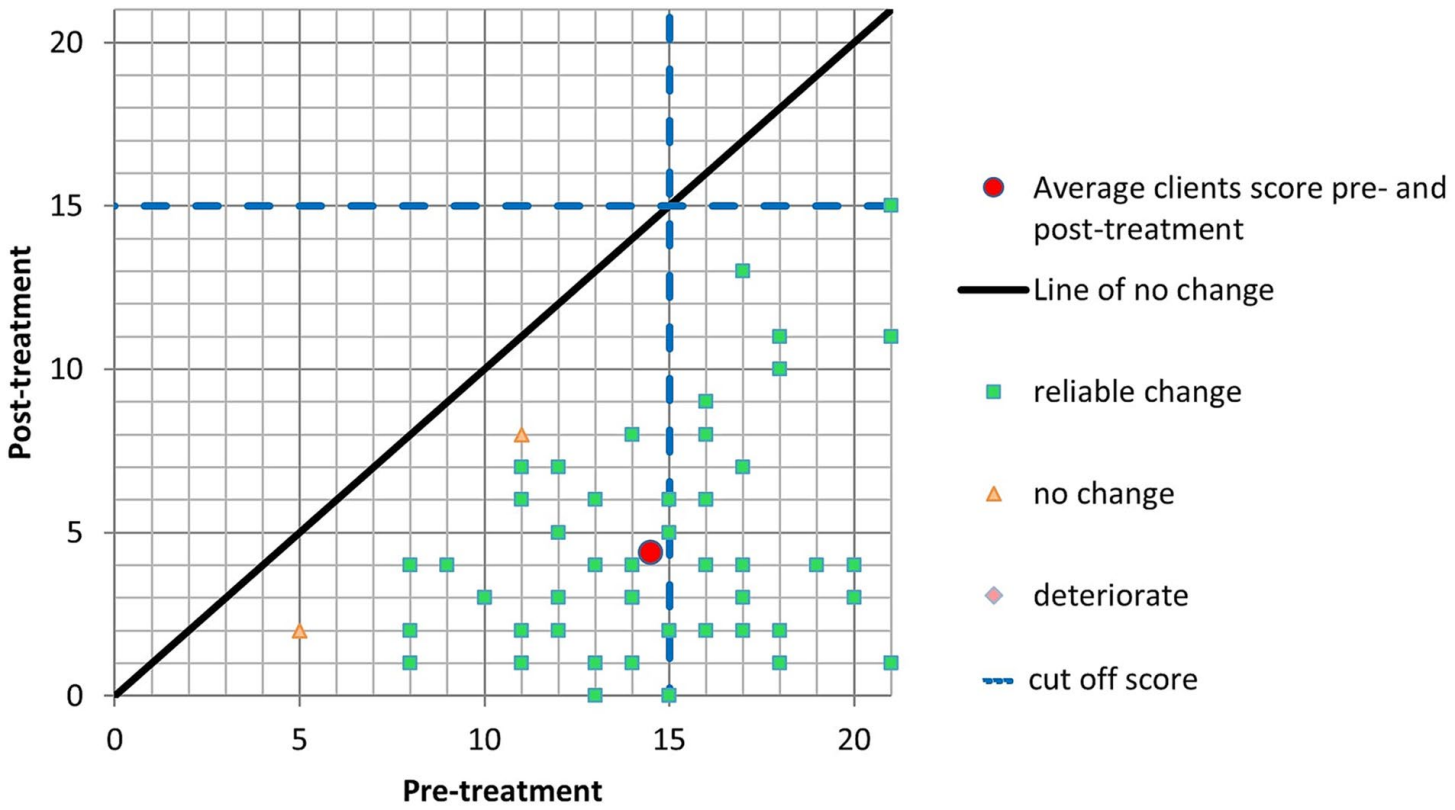
GAD-7.

Initially, patients had an average GAD-7 score of 14.51 (SD = 3.74) before treatment. Following the intervention, the average score decreased significantly to 4.38 (SD = 3.41). This reduction in anxiety symptoms indicates a significant positive change from pre-treatment to post-treatment. Furthermore, the Reliable Change Index (RCI), which assesses the statistical reliability of score changes, was calculated to be 3.28, suggesting a statistically significant improvement in anxiety symptoms. Fifty-one patients improved their GAD-7 scores after the intervention, reflecting a positive impact. Notably, no individuals experienced a deterioration in their symptoms, and three did not exhibit a significant change. In addition, a substantial portion of patients (50 individuals) met the criteria for Clinically Significant Change (CSC), indicating that the intervention led to a clinically significant reduction in anxiety (Figure 1).

**Figure 2:**
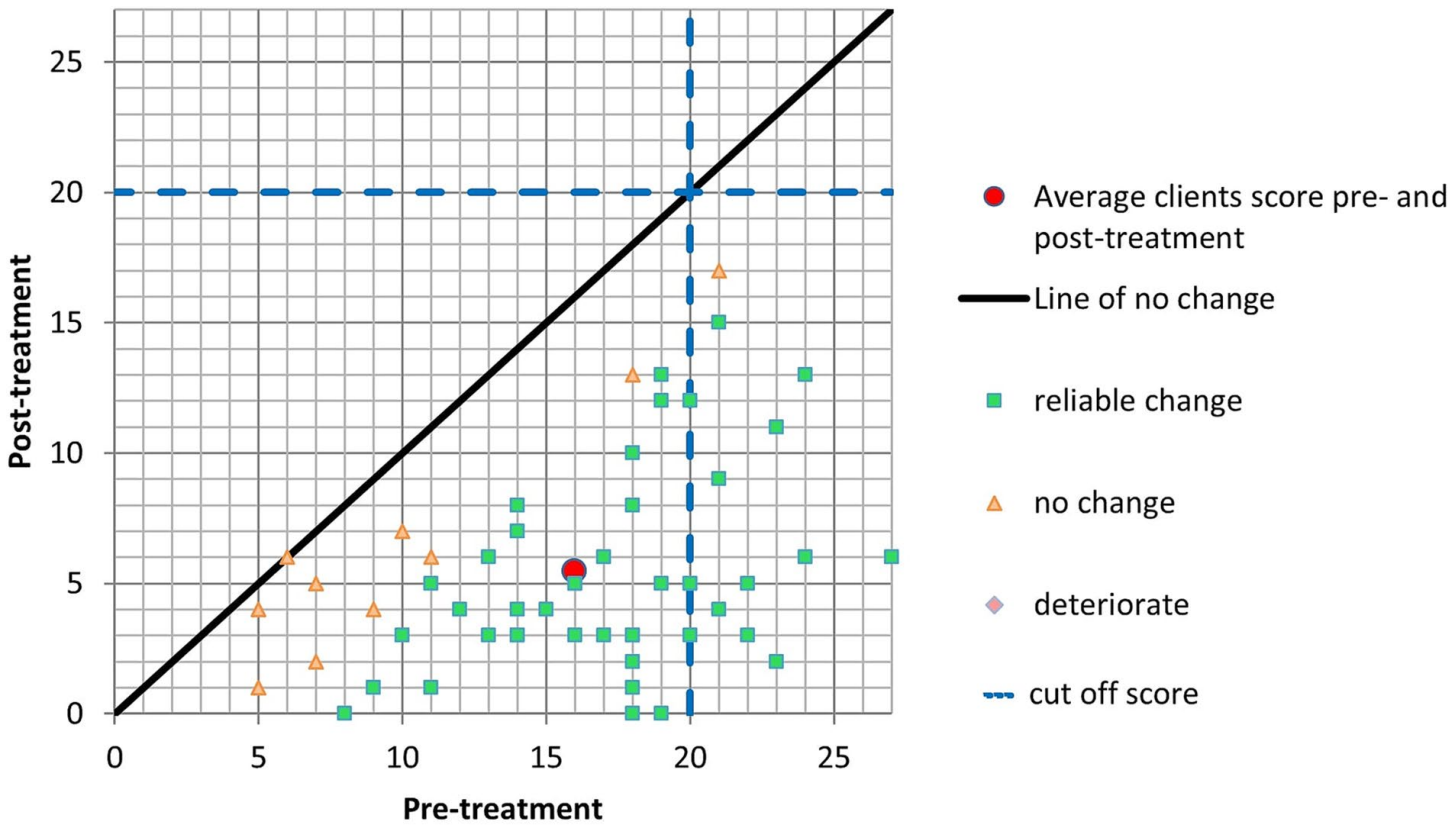
PHQ-9.

Initially, patients displayed an average PHQ-9 score of 16.15 (SD = 5.26) before the treatment. After the intervention, the mean score experienced a reduction to 5.53 (SD = 4.0). These values reveal a considerable and statistically significant change in depressive symptoms. Forty-four patients exhibited tangible improvement in their PHQ-9 scores following the intervention, indicating a positive therapeutic impact. Importantly, no individuals experienced worsening symptoms, and ten individuals did not manifest a significant change in their scores. Moreover, a substantial portion of patients (44 individuals) met the criteria for Clinically Significant Change (CSC), signifying that the intervention led to a substantial and clinically meaningful reduction in depressive symptoms (Figure 2).

**Figure 3:**
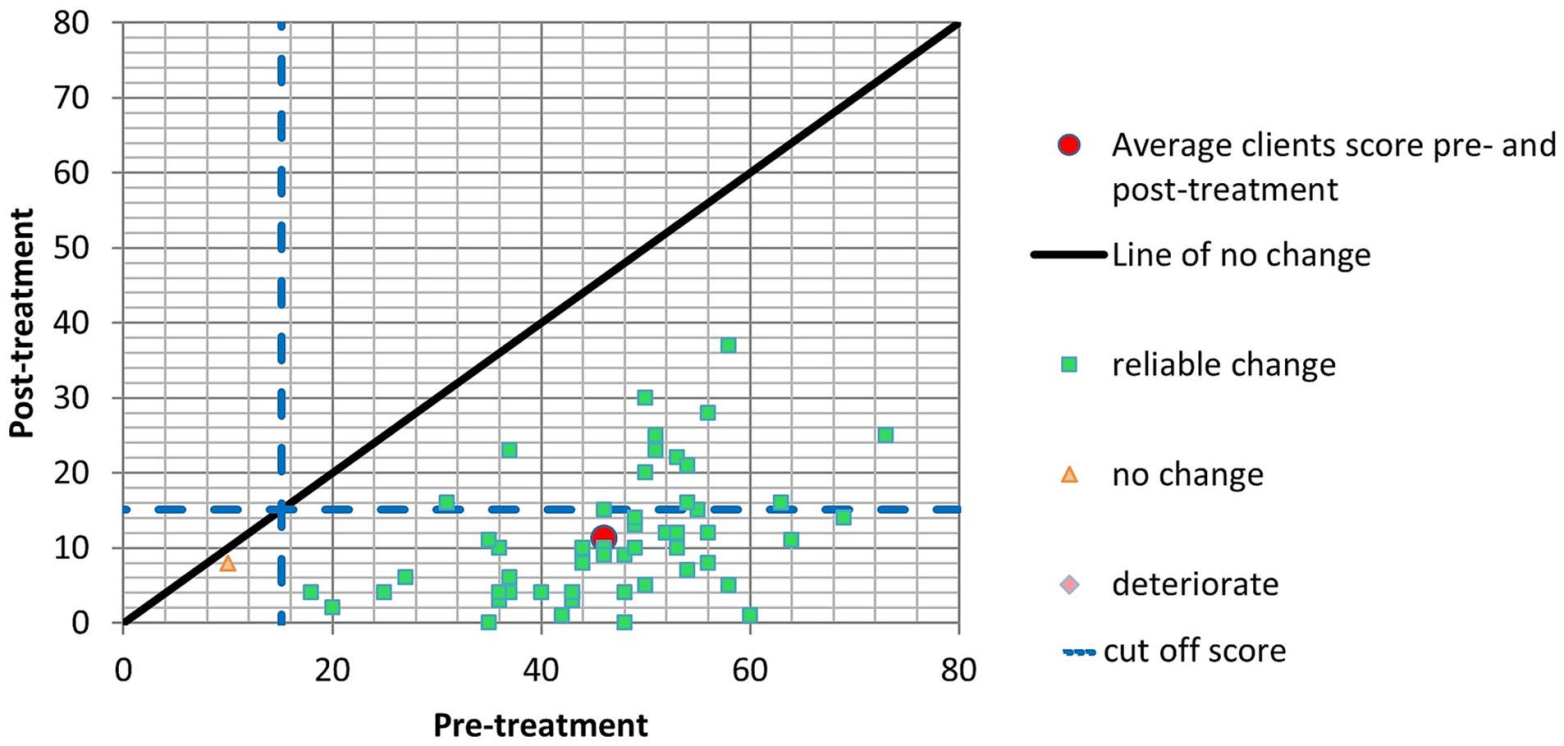
PCL-5.

Initially, patients exhibited an average PCL-5 score of 46.05 (SD = 12.33) before the treatment. Following the intervention, the mean score underwent a substantial reduction to 11.20 (SD = 8.39). This marked reduction in post-traumatic stress symptoms is underscored by a noteworthy pre-post effect size of 2.83, indicating a significant and positive alteration from pre-treatment to post-treatment. Furthermore, the Reliable Change Index (RCI) points to a statistically significant improvement in post-traumatic stress symptoms, as fifty-two patients demonstrated notable improvement in their PCL-5 scores following the intervention. Importantly, no individuals experienced worsening symptoms, and only two did not exhibit a significant change in their scores. Moreover, a significant number of patients (39 individuals) met the criteria for Clinically Significant Change (CSC), signifying that the intervention led to a clinically meaningful reduction in post-traumatic stress symptoms (Figure 3).

**Figure 4:**
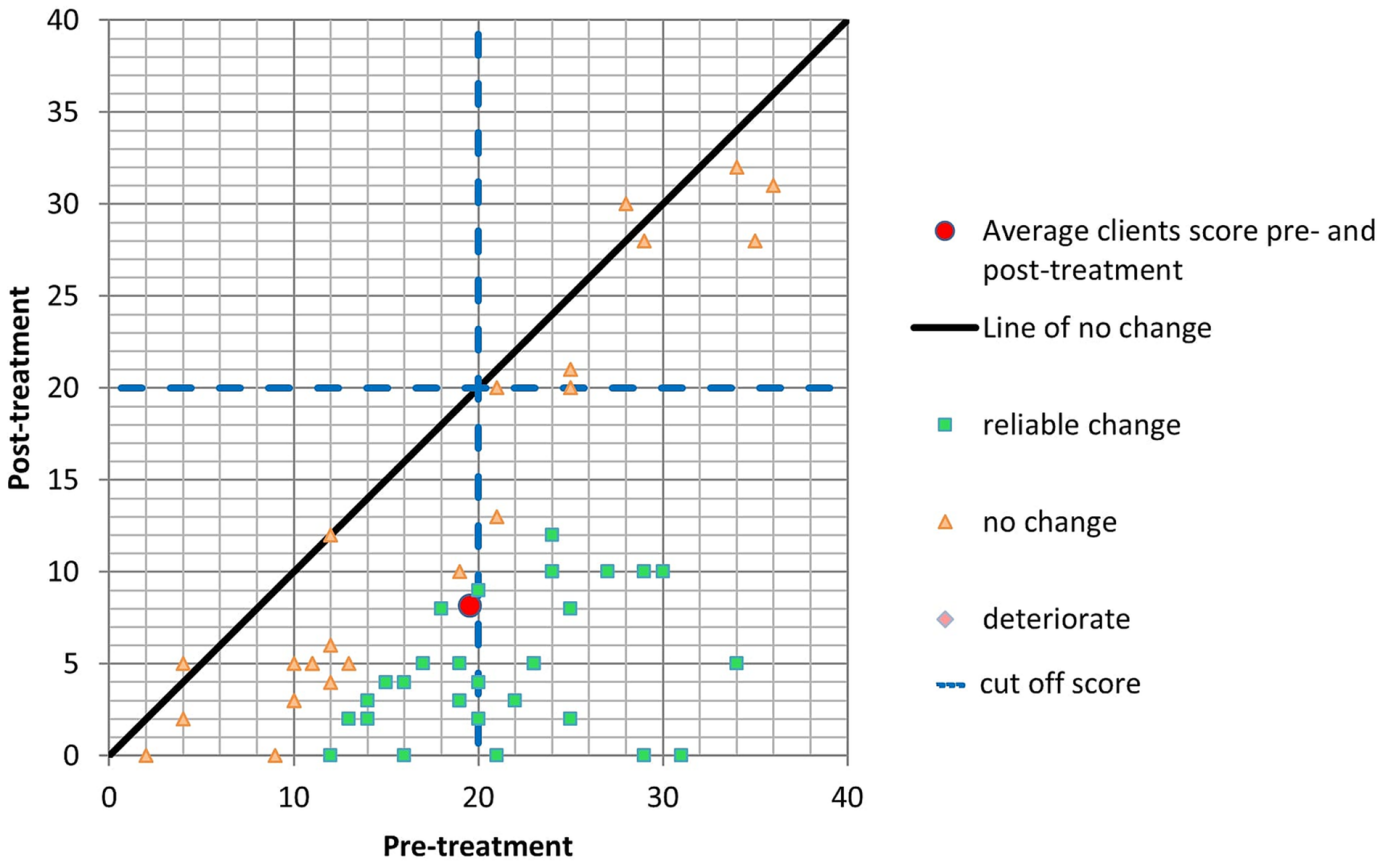
W&SAS.

Initially, patients displayed an average W&SAS score of 19.55 (SD = 8.24) before the treatment. Following the intervention, the mean score experienced a notable reduction to 8.15 (SD = 6.41). The Reliable Change Index (RCI) signifies statistically significant changes in symptoms. When examining specific outcomes, it is evident that thirty patients demonstrated substantial improvement in their W&SAS scores following the intervention, highlighting a positive therapeutic impact. Importantly, no individuals experienced a deterioration in symptoms, and twenty-three individuals did not manifest a significant change in their scores. Furthermore, a considerable number of patients (30 individuals) met the criteria for Clinically Significant Change (CSC), signifying that the intervention led to a clinically meaningful reduction (Figure 4).

### 3.3 Qualitative analysis

Sixteen patients and six GPs offered qualitative feedback via online questionnaires. Three themes with six subthemes were generated. A table of the themes can be found in Table 4.

**Table 4:** Themes and subthemes.

Theme 1: pleasantly surprised	Subtheme 1: “it has been a life saver”
	Subtheme 2: why it was effective
Theme 2: challenging but worthwhile	Subtheme 1: “going through memories is emotionally tough”
	Subtheme 2: “wish it was offered sooner”
Theme 3: what needs to be done next	Subtheme 1: some of us need more help
	Subtheme 2: offer it to other people and make it more mainstream

#### 3.3.1 Theme 1: pleasantly surprised

Many patients stated that they started with skepticism or doubts about EMDR but found the experience transformative. They described their experience with EMDR as “amazing,” “lifesaver” and “life-changing.” They described a reduction in their symptoms and a significant positive shift in their overall wellbeing, which is described in Subtheme 1. Patients also reported the reasons EMDR helped them, which is described in Subtheme 2.

##### 3.3.1.1 Subtheme 1: “it has been a life saver”

Many patients stated that EMDR had been highly effective for them. They reported experiencing a reduction in symptoms. These included but were not limited to a decrease in “fear” and “anxiety,” improved sleep such as ease in falling asleep, and a reduction in nightmares and night terrors. Some also mentioned EMDR’s positive effect on negative emotions.

Since the treatment I have been able to think about previous triggering events without negative feelings – Patient 6.

Some stated that on top of experiencing a reduction in symptoms, they also gained a different understanding and a change in perspective.

I was skeptical but halfway through my first session there was a breakthrough and lightbulb moment – Patient 5.

Some patients, on the other hand, took a step further, and explained that gaining this new perspective also enabled them to cope better outside of the therapy sessions.

It gave me an understanding of my own self, my thoughts and feelings. I learned coping strategies and feel able to handle situations that previously I would have run away from – Patient 11.

Some patients who had other forms of therapy before also noted that EMDR helped them process and manage their trauma more efficiently. Some also stated that EMDR had a relatively quick effect on them compared to what they had before.

I would never have imagined that such results could be achieved in such a relatively short amount of time – Patient 15.

Friends and relatives of those who underwent EMDR therapy observed positive changes in their emotional state.

This is obviously a subjective opinion, but friends and relatives have totally noticed the positive change in me. This is the best I have felt in 20 years – Patient 1.

GPs also highlighted that patients who have undergone EMDR therapy have experienced significant positive impacts on their lives.

I didn’t realize what a significant, positive impact it would be, having EMDR available within our PCN, when it was first commissioned. I now would be gutted if we ever lost the service! It has honestly transformed several of my patients’ lives and they couldn’t be more grateful for the help they got – Doctor V.

##### 3.3.1.2 Theme 1 subtheme 2: why it was effective

When discussing the reasons behind the effectiveness of EMDR for this patient population, GPs and patients agreed on the timeliness and promptness of this service. GPs emphasized that the availability of EMDR within the Primary Care Network (PCN) has prevented patients from experiencing prolonged waiting times for treatment, which could have led to worsening their conditions and increased burden on primary care services. They also highlighted that EMDR has helped prevent conditions from becoming chronic, which underscores the therapeutic value of EMDR in addressing issues early and preventing the long-term negative impact of untreated mental health conditions.

The EMDR service has been a great success. I have referred several patients who have received prompt assessment and successful subsequent treatment – Dr. N.

The timeliness of the EMDR intervention appeared to have been crucial and impactful for many of the patients who shared their experiences.

[EMDR was] very timely. Without it I am not sure I would still be here (alive) – Patient 5.

Some patients accessed EMDR therapy through referrals from their GPs (General Practitioners) or healthcare professionals. The referral process was generally seen as straightforward and facilitated by medical professionals. Many patients appreciated the accessibility and availability of EMDR therapy. They found it easy to connect with the service and received therapy relatively quickly and were offered flexibility with session times when necessary.

I found the service highly accessible, I was referred via my GP […] The EMDR sessions were timed well and there was flexibility when I needed it due to sickness or childcare issues. I felt the referral was appropriate given that I had already pursued talking therapy – Patient 6.

Some also compared the timeliness and promptness of PCN EMDR offer and their previous experiences when referred to mental health services.

Excellent response time. I have been referred to various providers in the past and this was by far the quickest response – Patient 2.

On top of appreciating the timeliness of the EMDR, some patients also commented on how straightforward this therapy is.

The process itself is straightforward to follow, my therapist answered questions with enthusiasm and passion – Patient 7.

Patients often mentioned the importance of the therapist-patient relationship, which could be one of the reasons why EMDR was effective for this population.

T [the therapist] was intuitive and professional. I was able to make a therapeutic relationship with her which ensured that I felt safe – Patient 6.

It seemed like the patients appreciated the therapist’s personability and treasured the therapist’s support, guidance, patience, and professionalism. According to the patients, their therapist made them feel at ease, provided a safe space, validated their feelings, and guided them through the EMDR process.

She [the therapist] was perceptive to my state of mind on the day and flexible with the format of the appointments. Her support and openness encouraged me to persevere – Patient 7.

[The therapy process was] upsetting at times however my therapist walked me through each step of the way and supported me – Patient 9.

#### 3.3.2 Theme 2: challenging but worthwhile

Although patients mostly talked about how the service they received was great and EMDR was efficient for them, they also shared the challenges they experienced during this journey. These are discussed in below two subthemes: Theme 2 Subtheme 1: “Going through Memories is Emotionally Tough” and ‘Subtheme 2: “Wish It was Offered Sooner.”

##### 3.3.2.1 Theme 2 subtheme 1: “going through memories is emotionally tough”

According to the patients, EMDR therapy was emotionally challenging, leaving patients emotionally and physically exhausted after sessions due to the intensity of processing traumatic memories.

The challenge came at the beginning of each session whereby I didn’t look forward to it as I knew it would stir up so many emotions – Patient 9.

Yes. I get very tired so it has been hard to put it into practice at times – Patient 14.

Additionally, some people talked about how opening up about deeply personal and traumatic experiences to someone else can be challenging.

I wanted to engage with the therapy 100% so had to be open and honest about what was going on in my life and what had happened. I found this very difficult as I had always kept it deeply buried and had always had an exterior protective shell that I presented to the world – Patient 15.

Patients seemed to create different strategies to better manage what comes with therapy. For example, a patient described how they put in extra effort to seek a good relationship with their therapist actively.

Talking openly about something that traumatizes you is not easy and for me it was really important to build trust and safety with a person. (…) I felt the need to build rapport through general conversation and I imagine this will vary from person to person. I certainly felt able to overcome my initial fears and feel more open discussing difficult subjects – Patient 6.

Some also stated that even though emotion processing was very hard for them initially, just practice and the passage of time also helped them cope better with processing memories and emotions.

It entailed driving through memories and events that caused me severe trauma and pain. However, as the process went on this became easier and was able to process these events still with great sadness and grief but without panic and or anxiety – Patient 13.

In addition to challenges that were brought by the therapy process, some also stated having problems with having the therapy online. While Zoom was well-received, some patients mentioned other platforms like Microsoft Teams as potentially more user-friendly alternatives. This likely reflects individuals’ feelings about the support and comfort they experienced while using Zoom for their EMDR therapy sessions. The consistent use of “Strongly Agree” might suggest a positive and supportive experience using Zoom for the therapy sessions.

##### 3.3.2.2 Theme 2 subtheme 2: “wish it was offered sooner”

Many people expressed gratitude that the EMDR intervention happened when it did. However, some stated it would have been better for their healing process if it had been offered sooner. For some, “sooner” could be measured by weeks:

It was good, on reflection I may of benefitted a little earlier- when I was initially off sick from work but I understand that there is a waiting list and was grateful for the opportunity – Patient 6.

However, others unfortunately seemed to wait longer than mere weeks, which seemed like a big challenge.

It has happened and for that I am so grateful. I wished it had happened years ago. It was the first therapy I had been offered rather than medication – Patient 1.

I consider myself extremely lucky that I got EMDR but it was such a long process with a lot of other options explored first. I presented years ago with PTSD symptoms and I do feel it should be offered sooner, in the first instance. If I had had EMDR off the back of the trauma 6 years ago I wouldn’t have had to live with it for years. I lost many years of my life just existing. This needs to be offered more often – Patient 16.

#### 3.3.3 Theme 3: what needs to be done next

The patients helpfully made recommendations to further improve the service they used. Their recommendations and comments were generated into two subthemes: Theme 3 Subtheme 1: Some of Us Need More Help is depicting how some patients explained that they required further sessions, while Theme 3 Subtheme 2: Offer It to Other People and Make it More Mainstream incorporates the importance of this service explained by both the patients and GPs, and how it would be more beneficial to improve and widen access.

##### 3.3.3.1 Theme 3 subtheme 1: some of us need more help

As stated before, many patients who received EMDR through their GP surgery explained they found it helpful. On the other hand, some stated that even though they benefited from the service, they thought they needed additional support. For some, this meant continuous additional sessions.

Just a shame it [the effect] wasn’t long lasting and that I now have PTSD symptoms again which started about two months ago – which was approximately three months after my EMDR sessions – Patient 3.

I personally struggled when my sessions ended so maybe more sessions were needed – Patient 12.

For others, extra help simply meant a single follow-up session, to check their progress and address if there are any emerging issues.

I think a follow up session after the initial therapy, conducted after a period of time to allow for grief and or trauma/stresses would be helpful/beneficial so that if any additional emotional/psychological interventions are required or even positive outcomes can be recognized and validated with a healthcare professional – Patient 13.

Some people stated that having in-person sessions rather than online could have been a better option.

Face to face may have been better but I have no complaints about it – Patient 10.

##### 3.3.3.2 Theme 3 subtheme 2: offer it to other people and make it more mainstream

Many patients desired to see the EMDR therapy service offered to more people through their surgeries. They believe that EMDR can be a game changer and should be accessible to a wider range of patients who could benefit from it. It seemed like they deeply empathized with others that might be suffering in silence,

We need more of it, I will be recommending this fantastic service to others I hope it becomes accessible to all appropriate people and I am extremely benefitted by receiving it – Thank you! Patient 6.

The patients also encouraged their healthcare professionals, such as GPs and nurses, to be more aware of EMDR therapy as an option and refer people who need it.

Making sure that surgeries are aware of the service offered and the suitability for issues that are covered – Patient 15.

Furthermore, some patients stated that EMDR should be offered more often, within and beyond the scope of the GP surgeries. They recommended increasing the mainstream visibility and integration of EMDR therapy to ensure awareness and access.

Make it more mainstream and make sure more people know about it. Without it, [I] may never have stood a chance of recovering – Patient 14.

Just like their patients, GPs also expressed hope and a desire for the PCN to continue investing in the EMDR service and to develop it. This demonstrates the GPs’ recognition of the service’s importance and their commitment to ensuring its availability to benefit more patients in the future.

Finding more practitioners as skilled as T [the therapist] to expand the provision of EMDR in the network would be very useful. Perhaps with a number of EMDR practitioners there may be scope for managing more complex PTSD patients – Dr. B.

Some GPs also highlighted what could have happened to their patients if the EMDR service was not offered, overlapping with their patients’ narratives around the importance of a timely and effective service for mental health difficulties.

Had this service not been available, these patients would still be on an NSFT waiting list, their conditions would have become chronic and much harder to treat, and they would likely have had far more contact with primary care services in the interim. I hope the service will continue to be supported by the PCN – Dr. N.

In conclusion, this thematic analysis highlights the positive impact of EMDR therapy while providing valuable insights into potential areas for enhancement and expansion. It is essential to consider these perspectives to continually improve and adapt mental health services to meet better the needs of patients seeking support.

## 4 Discussion

Untreated trauma can have a substantial negative impact on not only people’s mental health but also on their physical health (35). Research indicates that people with traumatic experiences not only can develop PTSD, but emotional trauma can also be a risk factor for many other mental health conditions, from eating disorders to psychosis (36, 37). Research on adverse childhood experiences also suggested that people with traumatic childhood experiences are more likely to develop physical illnesses such as stroke, cardiovascular disease, and cancer (38). Rising demand for mental health care, limited availability of psychological therapy, and an evolving understanding of the importance of trauma for psychological wellbeing are also causing frustrations for patients and primary care providers, thus forcing professionals to consider alternative methods and approaches. As one example, the present articles aimed to present the feasibility and potential effectiveness of remote EMDR therapy in enhancing the mental wellbeing of service users exclusively in primary care settings.

The findings of the EMDR in Primary Care Project presented here have exceeded our expectations and were recently shortlisted for the General Practice Awards 2022 in the Clinical Improvement: Mental Health category. As a result, our EMDR Consultant’s initial yearlong secondment from the mental health trust was extended to a further year and has now been converted into a substantive contract.

Overall, the results of the study indicated that, although not derived from a randomized controlled trial, a majority of clients who underwent EMDR observed improvements in their mental health, as evidenced by positive changes in scores on assessment tools. The reductions in anxiety and depression symptoms are particularly noteworthy, as these are common mental health challenges encountered in primary care settings. These findings are in line with the previous evidence where online EMDR was found to be an effective and promising intervention (20, 39–42).

Additionally, the qualitative analysis revealed that EMDR therapy was well-received and regarded as a favorable intervention by the patients. Patients’ descriptions of EMDR as “amazing” and “life-changing” highlight the transformational nature of the therapy. Importantly, patients linked these changes to gaining a new perspective, improving their mental health and coping better outside of sessions. Furthermore, patients discussed why EMDR worked for them: the straightforwardness of the EMDR therapy itself and the good therapist-patient relationship seemed to be what patients thought worked for them. Furthermore, the timeliness and the easy access of EMDR offer seemed to have a huge impact as many patients stated they favored the service for this reason. For that reason it is reasonable to believe that the promptness of EMDR therapy can play a crucial role in preventing conditions from becoming chronic, aligning with the patients’ and GPs’ endorsements of the timeliness of the service (4, 5). Patients’ positive shifts in behavior and improved relationships, as noted by friends and relatives, emphasize the broader positive impact on the patients’ lives and social functioning. While benefiting from EMDR, patients also acknowledged the emotional toll of EMDR therapy. However, they recognized the necessity of addressing these challenging emotions to achieve healing. Some patients also mentioned the taxing nature of therapy when conducted online, highlighting the potential benefits of in-person sessions.

Patients’ recommendations for further improvement and expansion of the EMDR service emphasize the desire for increased accessibility for themselves and others. They advocate for making EMDR therapy more mainstream and raising awareness among healthcare professionals, such as GPs, to ensure that more patients in need can access this effective intervention. Linked to this, patients often express frustration with the current healthcare system, citing the need to “jump through hoops” by undergoing multiple assessments and referrals. It seemed like they desire a simpler, more patient-centric approach to care. This includes a preference for a single, comprehensive assessment with a shorter waiting time. Access to care is also crucial as patients prefer therapy to be provided by their GP practice and addresses their needs allowing them to say, “X happened to me, and then symptom Y began.” Finally, such an approach not only benefits patients but also seems to relieve GPs of the moral injury they may experience when unable to provide fast or appropriate help to their patients.

The current evaluation shows that EMDR therapy in primary care services aligns well with patient expectations. It is known for its efficiency in addressing traumatic experiences and mental health issues (43). It typically involves a comprehensive assessment at the beginning of treatment, helping patients to connect their experiences to their symptoms, which fits the desire for a streamlined approach (44). It also has the potential to provide relatively rapid relief as some patients report experiencing positive changes in their wellbeing after just a few sessions, aligning with the desire for prompt results (45). Furthermore, EMDR can be offered in various healthcare settings, including those near GP practices, enhancing accessibility to care. Its patient-centered approach, where the therapist guides the patient through processing their traumatic memories, helps address the patient’s needs as they perceive them (46). These elements were pivotal in engaging and retaining service users within the project.

### 4.1 Implications and recommendations

Our findings underscore the potential of EMDR and online EMDR as an accessible and effective approach within primary care. The assessments showed an elevated level of access, completion and attendance among service users, which signifies a robust level of engagement and dedication to EMDR therapy.

Like other forms of psychotherapy, delivering EMDR remotely offers substantial advantages to clients. These benefits encompass a decreased need for travel time to and from appointments, elimination of income loss due to attending sessions, and reduced stigma often linked to seeking mental health treatment (47). Clients also gain greater control over their surroundings, and the use of desktop or smartphone applications that deliver alternating clicks through earphones can further empowers clients to manage their sessions effectively despite some cautions (48).

In collaboration with Primary Care Networks, Mental Health Trusts can use the model that has been described here to establish a highly responsive and accessible therapy service directly in GP practices. In the current project, the average time between the referral and the first contact was 15 days while the average time between the date of referral and the initial assessment was 26 days. Additionally, some clients requested a postponement of their EMDR sessions due to personal reasons, and they were able to begin their sessions at a time that suited them, showcasing both the responsiveness and flexibility of the service.

To further enhance the EMDR service, addressing the challenges associated with emotional processing, expanding access to face-to-face sessions, and reducing waiting times are crucial considerations. The positive feedback from patients and GPs underscores the importance of continued investment and development of the EMDR service within primary care services.

The positive clinical outcomes and patient testimonials highlight the potential for EMDR to become an integral part of standard mental health care within primary care networks. As more evidence of its effectiveness accumulates, healthcare systems may consider including EMDR as a recommended or first-line treatment option for specific mental health conditions. One of the key takeaways from this evaluation is the importance of timely access to EMDR therapy. The swift availability of EMDR prevented prolonged waiting times and according to the patients, helped prevent conditions from becoming chronic.

Continued research and evaluation are vital to understanding the long-term impact and potential refinements of EMDR therapy in primary care. Future studies can explore its effectiveness for specific populations, conditions, and in comparison, to other therapeutic modalities. Patients’ calls for making EMDR therapy more mainstream are significant. To achieve this, healthcare systems can consider integrating EMDR into broader mental health services and making it a standard offering for individuals experiencing trauma-related or other specified mental health difficulties.

### 4.2 Limitations

This evaluation and service come with several limitations. It is important to acknowledge that, despite significant interest, the dataset includes information from relatively few therapists. A small sample size of therapists might not provide a diverse enough range of perspectives, experiences, or practices, potentially limiting the broader applicability of the findings. Additionally, the absence of a control group prevents us from making any broad or generalized claims based on the findings. Moreover, as a service evaluation, this paper cannot give precise answers as to how providing EMDR in primary care affects morbidity, workload, or referral numbers nationally. Finally, due to the remote nature of the service and the limited number of sessions available, patients with dissociation were excluded. While dissociation commonly occurs after trauma and might exacerbate pre-existing conditions, this exclusion was implemented for the safety of the patients involved.

## 5 Conclusion

General Practitioners as family doctors enjoy a longitudinal view of their patients’ health. They can often observe first-hand the impact of traumatic events on their patients and the lasting effect on their lives. Yet, when they refer a patient with clearly trauma-related mental health difficulties, they have no influence over which trauma-focused therapy will be offered. Under present funding arrangements, GP’s can take matters into their own hands if they prefer not to wait until local provision of trauma-focused therapies catches up with patient needs: they can establish an effective service themselves. Thus, the future of EMDR therapy in primary care settings appears promising. Primary care networks and mental health trusts should consider expanding EMDR services, raising awareness among healthcare professionals, and integrating EMDR into standard mental health offerings. Tailoring support to individual patient needs and conducting ongoing research and evaluation will further enhance the effectiveness and accessibility of EMDR therapy.

## Data availability statement

The raw data supporting the conclusions of this article will be made available by the authors, without undue reservation.

## Ethics statement

Ethical approval was not required for the study involving human samples in accordance with the local legislation and institutional requirements because this is a service evaluation. Written informed consent for participation in this study was provided by the participants’ legal guardians/next of kin.

## Author contributions

SK: Data curation, Formal analysis, Methodology, Writing – original draft, Writing – review & editing. CD: Conceptualization, Funding acquisition, Methodology, Writing – original draft, Writing – review & editing. TD: Data curation, Methodology, Writing – original draft, Writing – review & editing. AA: Conceptualization, Formal analysis, Methodology, Writing – original draft, Writing – review & editing.
